# Explaining Residential Clustering of Large Families

**DOI:** 10.1007/s10680-023-09655-6

**Published:** 2023-04-19

**Authors:** Janna Bergsvik, Sara Cools, Rannveig K. Hart

**Affiliations:** 1 Statistics Norway, Research Department, Oslo, Norway; 2 Institute for Social Research, Oslo, Norway; 3 Norwegian Institute of Public Health, Oslo, Norway

**Keywords:** IV estimation, Neighborhood, Family size, Third births, Peer effects

## Abstract

Numerous studies have shown that fertility behavior is spatially clustered. In addition to pure contextual effects, two causal mechanisms could drive this pattern. First, neighbors may influence each other's fertility and second, family size may influence decisions about where to live. In this study we examine these two potential causal mechanisms empirically, using the sex composition of the two eldest children and twin births as instrumental variables (IVs) for having a third child. We estimate how having a third child affects three separate outcomes: the fertility of neighbors; the propensity to move houses; and the likelihood of living in a family-friendly neighborhood with many children. We draw residential and childbearing histories (2000–2018) from Norwegian administrative registers (N ~ 167,000 women). Individuals' neighborhoods are defined using time-varying geocoordinates for place of residence. We identify selective moves as one plausible causal driver of residential clustering of large families. This study contributes to the understanding of fertility and relocation, and to the literature on the social interaction effects of fertility, by testing the relevance of yet another network: that of neighbors.

**Supplementary Information:**

The online version contains supplementary material available at 10.1007/s10680-023-09655-6.

## Introduction

The spatial clustering of fertility is a well-established demographic finding. An extensive literature provides documentation that families have more children in rural than in urban contexts (Kulu, 2013). Furthermore, within urban regions families are consistently found to be larger in suburbs than in city centers (Kulu & Washbrook, 2014; Kulu et al., 2009). Evidence of the importance of local contexts for fertility behavior is also found for smaller geographic units such as city districts (Meggiolaro, 2011), statistical neighborhoods (Fiori et al., 2014) and couples’ nearest neighbors (Bergsvik, 2020).

Previous research suggests three important drivers of the residential clustering of fertility: First, neighbors share living conditions that are found to affect fertility—for instance, kindergarten supply (Rindfuss et al., 2010) and housing prices (Clark, 2012). Such shared conditions may give rise to *contextual effects*. Next, neighbors may influence each other’s fertility by exchanging information, norms and ideals. Such *social interaction effects* have been found among friends (Balbo & Barban, 2014), siblings (Lyngstad & Prskawetz, 2010; but for a counterexample see Cools & Hart, 2017), colleagues (Pink et al., 2014) and network members in general (Lois & Becker, 2014). Because neighbors and neighborhoods are an important part of families’ networks (Kalmijn, 2012), family size increases may influence neighbors’ fertility. Last, individuals may *self-select* into neighborhoods that fit well with their lifestyle and preferences, including their intended family size (Kulu & Washbrook, 2014). Couples who intend to have (many) children may prefer neighborhoods perceived as ‘family friendly’—e.g., with good schools, available green areas and spacious single-family houses (Mulder, 2013).

An extensive literature demonstrates a strong link between childbirth and residential relocation. As expected, the literature also finds that an increase in family size results in different housing needs. However, because residential moves are often made in anticipation of a birth (Ermisch & Steele, 2016; Öst, 2012), social interaction effects among neighbors are notoriously hard to distinguish from selective residential moves. For proper identification of both mechanisms, we need a research design that nets out confounding factors (Manski, 1995), i.e., one that nets out contextual effects on the one hand and clearly distinguishes between selective residential relocations and social interaction effects on the other. To the best of our knowledge, our study is the first to combine these goals in one study.

Our study further expands upon previous literature in three main ways. First, the literature on fertility social interaction effects and moving behavior has largely focused on the transition to parenthood or has not distinguished between first or higher-order births. The transition to parenthood entails distinct considerations, including questions of timing, compared to higher-order births in families who already have housing and some experience with kids. The focus of this study lies on family size and third births, an important margin in the Norwegian context. In 2018, 42 percent of Norwegian women at the age of 45 had given birth to two children and 29 percent had three children or more (Andersen et al., 2019).

Next, we add to this literature by exploring an alternative way of handling selection. We use random variation in the propensity of having a third child caused by two much-used instrumental variables (IVs) (Angrist & Pischke, 2009): twin births and sibling sex composition. We use the instruments separately, as the two IVs concern quite different fertility experiences (see also Sect. 3). A twin birth involves an unintended family increase, arguably random depending on the mother’s age (Black et al., 2005; Rosenzweig & Wolpin, 1980).^1^ Sibling sex composition is also random, but having two children of the same sex increases the probability of parents having a third child (e.g., Andersson et al., 2006; Angrist & Evans, 1998; Mills & Begall, 2010). Using these instruments, we net out contextual effects and test separately how family expansions influence residential choices and neighbors’ fertility.

Last, we contribute to the literature on network and neighborhood effects by operating with carefully constructed networks and neighborhoods (see also Entwisle, 2007). Using detailed geo-referenced data from Norwegian administrative registers covering the familial and residential histories of all residents of Norway for the years 2000 to 2018 (N ~ 167,000), we move beyond an understanding of families and their relocations as isolated actors and choices, instead recognizing them as being inherently linked to wider social contexts (Coulter et al., 2016). Moreover, rather than being viewed purely as fixed categories (such as ‘urban/rural’), our neighborhoods consist of the geographically closest inhabitants. In separate steps, we assess the dynamic neighborhood process, where families may influence a neighborhood, choose to relocate to a different one or stay in an already family-friendly neighborhood.

The country’s relatively high fertility and mobility makes Norway an interesting case for such a study. In our study period, the total fertility rate for Norway was shifted from 1.85 in 2000, to a high of 1.98 in 2009, and since declined to 1.56 in 2018 (Andersen et al., 2019). Due to Norway’s relatively low unemployment, high job protection and generous universalistic welfare state, individual families face little income insecurity and can rely on paid parental leaves of about one year for each child, as well as widely available public childcare for children above that age (Kravdal, 2016). About 80% of the population own their home, about 50% live in detached houses and only about 6% of households live in a crowded dwelling in 2021 (Statistics Norway, 2022). Yet, housing costs are high, especially in central regions, and housing costs represent more than 40% of the total disposable household income for about 10 percent of the population, which is close to the EU average (Eurostat, 2022). Relative to the rest of Europe, internal migration is high in Norway and the rest of Scandinavia, comparable to North America (Bell et al., 2018). Mobility is highest among young adults and in the establishing phase (ages 20–34), and short-distance residential moves dominate (Høydahl, 2022, see also Dommermuth and Klüsener, 2019). Despite relatively high mobility, national survey data reveal that 80 percent of couples with children at school ages know their neighbors well enough to visit each other occasionally, and that less than 10 percent of families find it hard to get help from neighbors (Statistics Norway, 2020). Together with Norway’s high-quality register data, these traits provide a promising ground for our analysis.

## Mechanisms of Spatial Clustering

### Social Interaction Effects Among Neighbors

For families with children, neighbors are quite strongly present in everyday life, whether it is at the local kindergarten, school or playground. Couples’ networks have been shown to shift to more local ties after they become parents, and respondents in a Swiss panel study state that they feel closer to more neighbors and report more neighborly contact and support after having a child than before (Kalmijn, 2012; Rözer et al., 2017). Parents have many opportunities to interact with neighboring parents, and such interaction might be particularly relevant. Neighbors may exchange knowledge and perceptions of norms, and through everyday interactions reveal the joys and stresses of life in families of different sizes. Through such social learning neighbors have the potential to shape what is seen as a normal or desirable number of children and in turn influence each other’s fertility behavior (Bernardi & Klärner, 2014).

Social interaction effects on the *transition to parenthood* have been documented for other peer groups than neighbors and might be present for increases in the number of children as well (see also Diaz et al., 2011). Individuals whose friends, acquaintances and siblings have young children are more likely to become parents, also when taking account of initial childbearing intentions (Lois & Becker, 2014). An individual’s probability of becoming a parent has also been found to increase after children are born to high school friends (Balbo & Barban, 2014), siblings (Lyngstad & Prskawetz, 2010), colleagues (Pink et al., 2014), a sibling’s colleague and colleague’s sibling (Buyukkececi et al., 2020). Pink et al. (2014) emphasize perceived similarity as an important amplifier of social learning effects, arguing that this should imply a social influence in respect of number of children.

Social influence among neighbors is examined for a range of individual outcomes such as mothers’ labor market participation (Maurin & Moschion, 2009) and problem behavior among adolescents, including early sexual activity (e.g., Browning et al., 2004; for a review see Sampson et al., 2002). Fertility contagion among neighbors, however, has mostly been studied in high-fertility contexts, for example, in rural Nepal (Axinn & Yabiku, 2001; Jennings & Barber, 2013), Kenya (Behrman et al., 2002) and Cairo (Weeks et al., 2004)—where individual fertility behavior and contraceptive use were found to vary with neighbors’ preferences and the local community context.

There is evidence that contextual factors such as community size and opportunity structures for families in a municipality also have a bearing on fertility behavior in countries that have already gone through major demographic transitions (e.g., Kravdal, 2002; Rindfuss et al., 2010; Vitali et al., 2015). However, no study has yet tested for the causal interaction effects of neighbors’ family behavior in a context such as Northern Europe, where fertility is usually seen as a highly individualized and couple-based choice (Lesthaeghe, 2010).

### The Effect of Family Size on Residential Adjustments

The actual or anticipated number of children may influence where couples want to live for several reasons. Most importantly, a larger family—all else equal—requires more space (e.g., Guest, 1972; Mulder, 2013). Furthermore, couples with more children may benefit more from living in a neighborhood with a family-friendly infrastructure than couples with fewer children. The value of access to good schools, recreational spaces and activities will increase with the number of children. Kulu and Boyle (2009) find supporting evidence in the form of selective moves from city centers to surrounding suburbs, which usually offer both more spacious housing and other family-friendly characteristics.

It is not surprising, therefore, that the propensity to undertake residential moves peaks around a new addition to the family (Ermisch & Steele, 2016; Mulder, 2013). Ermisch and Steele (2016) have also demonstrated how fertility intentions are a predictor of moves in Britain, indicating that couples move in anticipation of family expansions. In support of this, several studies find indications that moves precede (first) births (see Feijten & Mulder, 2002 for the Netherlands; Kulu & Steele, 2013 for Finland; Öst, 2012 for Sweden; Vidal et al., 2017 for Germany). In Norway, too, fertility intentions and migration intentions are positively related (Dommermuth & Klüsener, 2019). In addition, however, transition into parenthood and growing family size are found to be associated with a lower propensity to make (long-distance) moves (Clark & Withers, 2007; Dommermuth & Klüsener, 2019; Kulu & Milewski, 2007; Long, 1972). Ermisch and Steele (2016) discuss a ‘taste for stability,’ where individuals with more children are less likely to relocate due to the high cost of moving with a large family and families’ place attachment, e.g., the importance of local networks for parents and children, as well as possible established ties to local schools and kindergartens (Clark et al., 2017). By using exogenous increases in family size we are able to study how an unanticipated third birth affects residential adjustments (see also de Groot et al., 2011).

As housing and childbearing decisions (and plans) are often made together, they can be jointly influenced by values and ideals (‘tastes’), but of course both also be enabled or constrained by financial resources. Kulu and Steele (2013) model residential moves and childbearing jointly and find that the two processes are positively correlated, i.e., that individuals prone to relocate are also more likely to have children. This simultaneity complicates assessing whether childbearing has a causal effect on residential moves. Because (long-term) fertility intentions can influence residential decisions (Ermisch & Steele, 2016), a correct temporal ordering of events is not sufficient to provide proof of causality.

## Self-Selection and Confounding Factors: The Scope for Using IVs

In order to empirically identify the separate mechanisms of social interaction effects and relocation behavior, we need to distinguish them both from each other and from confounding factors and other forms of self- selection. Using a source of exogenous (random) variation in family size could potentially solve these problems.

With regard to social interaction effects, two main factors complicate the task of causal identification: In addition to being influenced by each other, neighbors may display similar behavior because they are similar at the outset (which, in turn is the result of selective residential sorting) and/or because they are influenced by the same environment (contextual effects). An exogenous source of variation in fertility would be independent of both the self-selection of neighbors and their shared environment. Hence, evidence of social interaction effects exists if an exogenous increase in the family size of one neighbor tends to be followed by a change in another neighbor’s fertility.

With regard to estimating the effects of larger family sizes on residential relocation, self-selection may be a confounder, albeit in a slightly different way. Consider two couples, one residing in a large suburban house with four children, another in a compact central urban apartment with one child. Surely the number of children need not be the only difference between the couples of relevance to their residential decisions. Differences in taste and lifestyle preferences, in combination with economic resources, are likely to influence both residential decisions *and* fertility decisions (see also Bruch & Swait, 2019; Schachner & Sampson, 2020). Again, we want to isolate the effect of family size alone on residential decisions, using an exogenous source of variation in family size.

One approach to handling the simultaneity of housing and fertility decisions has been to model the two processes jointly within a multilevel, multiprocess statistical framework. Kulu and Steele (2013), for instance, find that results change little when housing and fertility decisions are estimated simultaneously, with their residual effects allowed to correlate. However, this modeling strategy will not handle omitted variables. Estimates are therefore prone to suffer from omitted variable bias (Wooldridge, 2010). To further improve the understanding of the drivers of the residential clustering of large families, this paper tests another approach, using instrumental variables.

We apply two much-used instrumental variables in order to obtain exogenous variation with respect to having a third child: a twin birth at second parity (Rosenzweig & Wolpin, 1980) and the sex composition of the first two children (Angrist & Evans, 1998). Twin births represent an unplanned immediate increase in family size and a permanent increase in family size for couples who would otherwise not have had more children. To the extent that having twins is conditionally random (i.e., if parents of twins are no different from parents of singletons after observable characteristics are netted out), it is potentially valid as an IV for family size. The sex-composition instrument relies on the fact that many couples prefer having one child of each sex, so that they will have a third child if and only if the first two are of the same sex (Andersson et al., 2006). As child sex is random, so are increases in family size induced by sibling sex composition. Tests for (conditional) randomness of observable characteristics are presented in Sect. 4.5.

The two IVs employed in this paper represent quite different fertility experiences. The twin instrument captures the effect of a third child among couples who would otherwise have preferred only two children, whereas the sex-composition instrument captures the effect of a third birth among parents who would have stopped at two children if—and only if—they were of different sexes (see also Cools et al., 2017; Hart & Cools, 2019). For many reasons, having another child because of a desire for children of both sexes could be less demanding than having twins. Most importantly, there is no spacing between twins, which might make the family increase more stressful both practically and economically.

In order to be valid, our instruments must affect our outcomes through the instrumented variable (family size) only. This assumption cannot be tested directly but must rather be approached through reasoning and indirect tests (e.g., Huber, 2015). With regard to the twin instrument, the short spacing itself ought to have no direct effects on neighbors’ fertility and the family’s residential decisions. When it comes to sex composition, earlier research has argued that children of the same sex generate less expense because, for example, they could share clothes and a room, which could lead to different effects on relocation behavior compared to that of families that are otherwise of the same size. However, differences in the economies of scales attributable to children’s sex composition have not been confirmed for high-income countries (Huber, 2015).

It might be easier to compare the effects of average third births in the population to the effects of a third birth resulting from a preference for having one child of each sex, than to the effects of a twin birth at second parity. A twin birth represents an unplanned shock and results in an ‘extra’ child for a much larger share of the population. While our main aim is to test which of the mechanisms behind the spatial clustering of large families we find causal evidence for, we also explore the different natures of the fertility shocks represented by the two instrumental variables, in order to see what bearing they have on interaction effects and residential decisions.

### Hypothesis

As outlined above, previous research has shown that peers influence each other’s fertility. In the Norwegian context, a high percentage of the population has contact with their neighbors and the third child represents an important choice margin toward a ‘typically larger’ family. We therefore expect that neighbors might represent such peers and hypothesize:

(A) Having a third child causes one’s neighbors to have more children

Arguably, a neighbors’ third birth carries more relevant information for mothers of two children, for whom a third child constitutes the next choice margin. We explore empirically whether neighboring women with two children are more easily influenced. At the same time, births often trigger residential adjustments and there is relatively high residential mobility in the Norwegian population. We therefore expect:

(B) Having a third child causes one to relocate

Mothers of three children may relocate to a more family-friendly neighborhood, and/or to a more suitable dwelling in their current neighborhood. To explore the relevance of each of these types of moves, we separately estimate effects on short and long moves, and we investigate whether mothers who were living more cramped at the outset are more likely to make such moves (and to make them more immediately).

Finally, families with three children may move to a more family-friendly neighborhood, or be more likely to stay if they live in one. We hypothesize:

(C) Having a third child makes one choose family-friendly neighborhoods

We test whether having a third child increases the likelihood of living in neighborhoods with many children and in addition whether living in a neighborhood with particularly many large families is more likely.

## Data and Methods

This study is based on combined individual-level records from several Norwegian administrative registers covering residential and childbearing histories for the whole population of Norway in the years 2000 to 2018. Women are linked to their neighbors and children by means of annually updated geocoded addresses and personal identification numbers (PINs), respectively.^2^

### Study Sample and Timeline

**Fig. 1:**
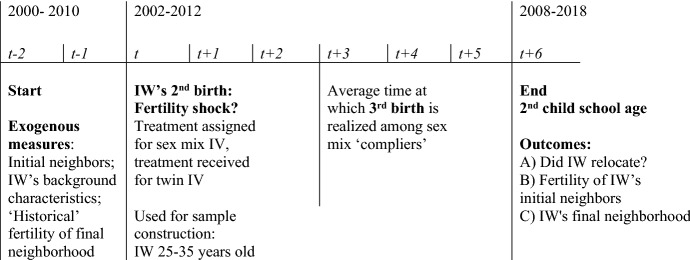
Timeline for measurement points in study

Our *study sample* consists of women who gave birth to a second child between 2002 and 2012, represented by measurement point ‘t’ on the timeline in Fig. 1. Inclusion in the sample was conditional on being aged between 25 and 35 at the time of the second birth and being registered with a Norwegian address two years before the birth, i.e., in t-2 (~ 167,000 women). Having their relevance as peer group for other mothers in mind, we have limited the sample to the age window in Norway when it is most normal to have the second child while at the same time not being too near the end of the fecund period. The latter is important regarding the use of the instruments: The likelihood of having problems to conceive increases with age, and such problems threaten the validity of both instruments.

To analyze how an increase in one of these women’s fertility affects her neighbors’ fertility, we split the sample into two parts: A 33% random subsample of these women (~ 55,000) constitutes the ‘index women’ (IW) whose fertility will potentially influence the fertility of their neighbors. The remaining 67% enter the pool of neighbors whose fertility is potentially influenced by that of the index women. Thus, no pair of women can mutually influence one another—every woman is either a potential influencer or potentially influenced, thereby preventing a reflection bias (Manski, 1993).

Index women’s *individual neighborhoods* are captured two years before the birth of their second child (2000–2010, t-2 in Fig. 1) and are defined by means of geographical coordinates for place of residence at the end of that year. Neighborhoods consist of each woman’s 50 nearest female neighbors aged 20–44, defined by straight-line distances. Within the neighborhood, women aged 20 to 36 at start are defined as ‘potentially influenced.’ This gives on average 29 neighboring women, of whom 15 are mothers, six of them with two children. The average distance between the index women and their neighbors is approximately 400 m (median approximately 136 m) (see Appendix Table A1).

### Outcome Variables

In several separate models, we analyze how a family increase (i.e., having a third child) influences three types of outcome (see also Table 1):

(A) The fertility of neighbors,
(B) The propensity to move,
(C) Characteristics of the final neighborhood.

As can be seen in Fig. 1, we measure these outcomes six years after the birth of the index woman’s second child (at measurement point t + 6), i.e., when the neighboring women are 28–44 years old, index women’s twins are six years old and the third child of the mean (median) ‘complier’ is 2.2 (2.7) years old. This time frame balances the need for a sufficiently short time lag after the exposure while at the same time giving families some time to realize their housing and fertility desires.^3^

#### Fertility of Neighbors

Our first outcome is the average number of children among young female neighbors from index women’s initial neighborhood. Besides measuring the aggregated number of children at the end of our observation period (in year t + 6), we distinguish between those who at start (in year t-2) were either i) childless women, ii) mothers with one child or iii) mothers with two children.

#### Propensity to Move

To measure the propensity to move, we construct an indicator variable taking the value ‘1’ if the mother has moved at least once between the year before the second birth (t-1) and the year the second child turns six (t + 6), otherwise zero. Since we are also interested in the distance of the move, we additionally estimate whether the mother has had a move of at least three kilometers.^4^

#### Characteristics of the Final Neighborhood

The last outcome captures aspects of the neighborhood where the index woman lives when her second child is six years old (measurement point t + 6 in Fig. 1)—independently of whether she has moved or not. We proxy the ‘family-friendliness’ of the final neighborhood by the average number of children per woman aged 25–44 in that neighborhood—as measured eight years earlier.^5^ We measure these characteristics eight years earlier (in t-2, two years before second birth) in order to construct a measure that is free from potential interaction effects running from the index woman to her neighbors. To explore effects at different margins, we also construct variables that capture the proportion of women in the neighborhood eight years earlier that had at least one, at least two and at least three children.

**Table 1:** Descriptive statistics for outcomes

	Statistics		
Outcome variable	Mean	SD	N
*Fertility of neighbors*^a^			
Young female neighbors’ no. of children in t + 6	1.613	0.351	54,787
Previously childless neighbors’ no. of children in t + 6	0.847	0.301	54,755
1-child neighbors’ no. of children in t + 6	1.809	0.385	54,475
2-child neighbors’ no. of children in t + 6	2.344	0.312	53,461
*Propensity to move*^b^			
Moved by the time 2nd child is 6 years old	0.661	0.473	166,710
Moved at least 3 km	0.423	0.494	166,927
*Characteristics of the final neighborhood*^c^			
Average number of children per female neighbor 25–44	1.394	0.303	166,666
Percentage with at least one child	64.93	11.05	166,657
Percentage with at least two children	48.18	11.82	166,657
Percentage with at least three children	18.06	8.41	166,657

^a^ Young female neighbors who were 20–36 years old and living next to index woman in t-2

^b^ Cumulative sum measured from the year before IW’s second birth (t-1) until six years after (t + 6)

^c^ Neighborhood where family lives when 2^nd^ child is six years old (t + 6). Neighbors’ characteristics measured at start (t-2). Here, neighborhoods refer to basic statistical units with on average 131 women of age 25–44

### Background Characteristics

To increase precision and to meet the assumption of (conditional) random assignment (for the twin instrument), we include several observable characteristics of index women and wider geographical attributes of neighborhoods as covariates in all regression models. We also include calendar year dummies in all models.

*Individual characteristics* include mother’s age the second time she gives birth, the time in years between the first and second births (min. 0.75 year = nine months) and an indicator for being foreign-born (the reference case is Norwegian-born.) A mother’s employment status was defined as active (ref.) if her annual wage income exceeded the social security base income (~ 50,000 NOK in 2000, ~ 6,000 $). Her income (inflation-adjusted to 2000-NOK) is included in terms of her position in the sample’s income quartile (Q1: NOK 135,000; Q2: NOK 215,000 (ref.); Q3: NOK 275,000). A set of dummies for educational attainment distinguishes between the following categories: (i) Primary education (≤ 10 years); (ii) Secondary education (11–13 years) (ref.); (iii) Short university education (14–17 years); and (iv) Long university education (≥ 18 years). We also include a covariate for time since last move, measuring the number of years a mother has lived in her current dwelling, including a squared term to capture possible nonlinearities. All characteristics are measured two years before the second birth (in t-2).

*Place of residence* is captured by a set of dummies for the seven main regions in Norway which are: the Capital region (previously Oslo and Akershus, ref.), South Eastern Norway, Hedmark and Oppland (now: Innlandet), Agder and Rogaland, Western Norway, Trøndelag, and Northern Norway. Further, a measure of municipal centrality is included. Centrality describes a municipality’s population size and geographical position in relation to urban areas (see Statistics Norway Standard Classification of Centrality at http://stabas.ssb.no/, 2014 classifications). This study used the following five categories: (i) Municipality with a regional center; (ii) Municipality within 35 min commuting time of a regional center (ref.); (iii) Municipality within 36 to 75 min commuting time of a regional center; (iv) Somewhat central municipalities; and (v) Less and least central municipalities.

*Housing characteristics.* Housing data have been retrieved from the official registry of ground properties and addresses and are linked to individuals through detailed address codes.^6^ For housing type, we differentiate between apartments and (row) houses. For number of rooms in current dwelling, we distinguish between those with (i) up to four and (ii) at least five rooms (excluding kitchen and bathrooms).

### Statistical Model

The IV estimation is done in two steps, using 2SLS regression. The two stages are modeled as follows:

First stage:

1 $${x}_{i}= \delta {\overline{w} }_{i}+\rho {z}_{i}+{\epsilon }_{i},$$

where ${x}_{i}$ is and indicator variable for whether individual $i$ has a third child; ${\overline{w} }_{i}$ is a vector of control variables; and ${z}_{i}$ is the instrumental variable indicator—taking the value 1 if individual $i$ has two children of the same sex or twins at second birth, respectively. $\rho$ is the first-stage coefficient on the instrumental variable.

Second stage:

2 $${y}_{i}=\alpha {\overline{w} }_{i}+\beta {\widehat{x}}_{i}+{\epsilon }_{i},$$

where ${y}_{i}$ is the outcome of interest, ${\overline{w} }_{i}$ denotes the same set of control variables as in Eq. (1), and $\beta$ is the 2SLS estimate of how having a third child influences outcome ${y}_{i}$.

First, we obtain the first-stage estimates in Eq. (1), which estimate the effect of having twins or of the two eldest children being of the same sex on family size—captured by the probability of having a third child before the second child reaches the age of six. In the second stage, IV estimates are obtained by regressing the outcomes on the part of the variation in family size that is linked to twins or sibling sex composition (Eq. (2)). The IV estimates capture the average effects on those influenced by the instruments (‘compliers’)—that is, those mothers who will have a third child if and only if the second birth produces twins, or the first two children are of the same sex (Angrist & Evans, 1998). We also present reduced-form estimates of the effect on the outcomes of sibling sex composition or of having twins. The reduced-form estimates capture how a twin birth or sibling sex composition affects the outcome in question, without assuming that the effect is channeled through family size. Last, we give estimates of the correlation between outcomes and family size using OLS regression. All specifications include dummies for calendar year and mother’s age at the time of the second birth.

### Descriptive Statistics and Balancing Tests

In order to see whether the instrumental variables we use are randomly assigned, we test differences in the background variables of mothers according to whether they had either twins at the second birth or a second child of the same sex as the first, or neither of these. The results of these tests are shown in Tables 2 and 3. For the instrument to be randomly assigned, there should be no systematic differences by instrument status on outcomes measured before the instrument is assigned.

**Table 2:** Background characteristics by instrument status (full sample)

				Singleton birth		
	*Twin*	*Singleton*	T-test	*Same sex*	*Diff. sex*	T-test Diff
	Mean	Mean	Diff	Mean	Mean	
Covariates	(1)	(2)	(3)	(4)	(5)	(6)
Birth year 2nd child	2004.91	2005.02	.108	2005.01	2005.03	.021
Age at 2nd birth	30.929	30.331	− .597^***^	30.338	30.324	−.014
Years since 1st birth	4.049	3.686	− .363^***^	3.681	3.692	.009
Norwegian born	.894	.861	− .033^***^	.860	.861	.001
N	2,771	164,156	166,927	81,948	82,208	164,156

^***^p < .001

**Table 3:** Balancing tests: unconditional and conditional dependence on IVs (reg X IV)

	IV = twin		IV = same sex	
X	(1)	(2)	(3)	(4)
Active in labor force	.013^*^	−.003	−.000	−.000
Income in 1000s of NOK	10.121^***^	2.461	1.012	.893
Has higher education	.020^*^	.004	.002	.001
Time since last move	.242^***^	.131^*^	.005	.005
Table 2 covariates included	No	Yes	No	Yes
N	166,927	166,927	164,156	164,156

Covariates include: birth year 2nd child, mother’s age at 2nd birth, years since 1st birth and Norwegian born

^†^*p* < .1; ^*^p < .05; ^**^p < .01; ^***^p < .001

For the sex-composition instrument (column 4–6), there are no significant differences by instrument status. Mothers whose first two children are of the same sex are statistically similar to mothers whose two first children are of opposite sexes with respect both to age, years since first birth and having been born in Norway. For the twin instrument (column 1–3), we find multiple statistically significant differences by instrument status, some of them of sizeable magnitude. This finding is in line with previous applications, which show this instrument to be only conditionally random (Hart & Cools, 2019).

To test whether twin births are conditionally random in this sample, in Table 3 we estimated how the IVs predict several other background characteristics, first without conditioning on the background variables in Table 2 (columns 1 and 3), then conditioning on them (columns 2 and 4). Under conditional independence, significant associations should disappear when background characteristics are controlled for in Table 2 (see also Hart & Cools, 2019). For the sex-composition instrument, there are no significant associations. The twin instrument is significantly associated with the outcomes in Table 3, but the association disappears for all characteristics except time since last move when covariates are included in column 2. In order to account for these differences, we include these covariates as controls in the analyses.

## Results

### Social Interaction Effects Among Neighbors

In this section, we test Hypothesis A: that having a third child causes one’s neighbors to have more children themselves. The main estimates of these social interaction effects are presented in Table 4. In all tables, the even-numbered columns also include a set of exogenous control variables (see Sect. 4.3). The upper and lower panels give estimation results for the twin (Panel A) and the sex-composition (Panel B) instruments.

**Table 4:**
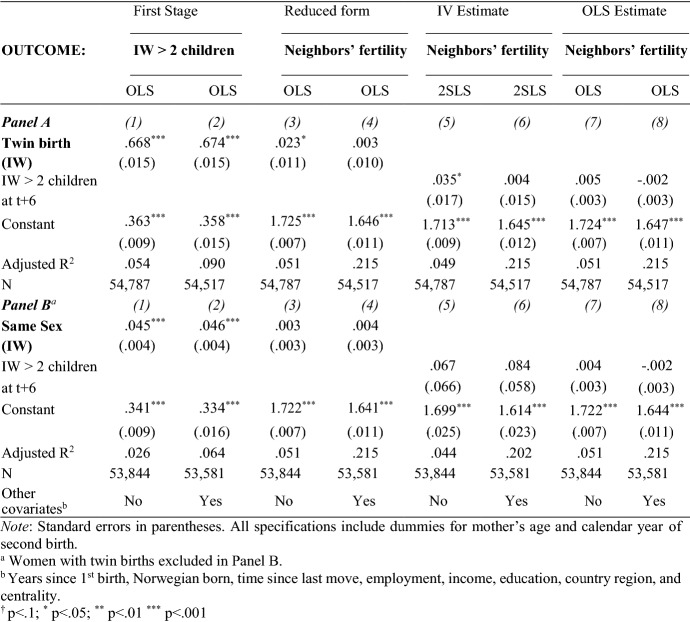
Effects of index woman’s twin birth, sibling sex composition and third child on initial young female neighbors’ average number of children (in year t + 6)

#### First-Stage Estimates

Having twins raises our sample mothers’ probability of having three children by 67 percentage points on average, meaning that 33 percent of mothers would have had a third child within six years anyway (first-stage estimates, columns 1 and 2 in Table 4). Having two children of the same sex increases the likelihood of having a third child (within six years of the second birth) by about five percentage points. The F-statistics are above 10 for both instruments (not shown), thereby satisfying the criterion for instrument relevance.

#### Main Results

To measure the general effect of index women’s fertility on their neighbors’ fertility, we use as an outcome *the average number of children among neighbors six years after the index woman’s second child is born*. The results indicate that the index women’s fertility shock has no significant effect on neighbors’ fertility when instrumented with twin births or the first two children’s sex composition. Interestingly, the OLS estimates show that an index woman’s third birth is not even correlated with her initial neighbors’ future numbers of children.

We expected that a neighbor’s third birth may carry most relevant information for mothers of two. However, dividing initial neighbors into subgroups by their number of children at the start (t-2) does not reveal any effects among neighbors with two children at the onset (see Appendix Table A2).

### The Effect of Family Size on Residential Adjustments

This section tests Hypothesis B that mothers relocate because they have a third child. The main results for residential adjustments are presented in Table 5. The control variables are identical to the ones used before. The first-stage estimates turn out to be identical, also in this larger subsample.

**Table 5:**
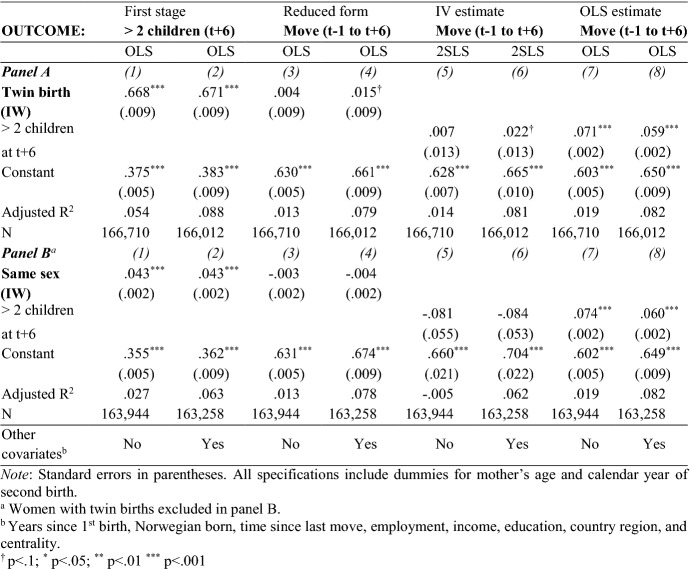
Effects of a twin birth, sibling sex composition and having a third child on propensity to move

#### Main Results

The IV estimates in Table 5 show that, instrumented with a twin birth, having a third child increases mothers’ probability of moving within six years of the second birth by 0.022 (*p* < 0.1), on average (column 6).^7^ Conversely, the estimates derived from the sex-composition instrument are negative and not statistically significant. The OLS estimates show, consistent with previous research, that having a third child is positively correlated with a mother’s propensity to move (columns 7 and 8).^8^ The OLS estimates are substantially more positive than the 2SLS estimates in column 6.

#### Mechanisms

Our main results show how having a third child affects mothers’ propensity to move at least once in the period between the year before the second birth and six years after the birth. Further analysis of the distance of moves shows that these are driven by a higher propensity to relocate in the immediate neighborhood (Appendix Table A3). There are no effects on relocations of three kilometers or more.^9^

To the extent that the effect of the (twin) fertility shock on moving is driven by the need for more housing space, we expect the effects to be stronger—and more immediate—among those that started out with relatively smaller dwellings.^10^ In Appendix Fig. A2, we show yearly estimates for different subsamples depending on dwelling size (number of rooms) and type (house or apartment). The twin IV estimates confirm that immediate effects are concentrated among mothers in relatively smaller dwellings (with up to four rooms) and mothers in apartments. Mothers in apartments are more likely to move all measured distances right after a (twin) fertility shock and the effect persists for moves further than three kilometers (results upon request). For mothers who start out in houses, immediate effects are smaller, but they persist.

To sum up, our analyses show that a family increase due to the desire to have one child of each sex does not significantly affect the relocation behavior of mothers after they give birth to a second child with the same sex as the first. On the other hand, a family increase due to a twin birth increases a mother’s propensity to relocate, driven by short-distance moves and with strongest immediate effects among women in smaller housing. This points to that the need for space triggers a residential adjustment.

### The Effect of Family Size on Characteristics of the Final Neighborhood

Last, we test hypothesis C: that having a third child affects one’s likelihood of living in a family-friendly neighborhood, measured six years after the second birth. Importantly, we do not consider whether mothers move or not, meaning that family size can affect neighborhood characteristics both by inducing and preventing moves. The main results are presented in Table 6. The sample and control variables, as well as the first-stage estimates (see columns 1 and 2), are identical to those in Table 5.

**Table 6:** Effects of a twin birth, children’s sex composition and having a third child on average number of children in the final neighborhood

	First Stage		Reduced form		IV Estimate		OLS Estimate	
**OUTCOME:**	**IW > 2 children (t + 6)**		**Average no. of children**		**Average no. of children**		**Average no. of children**	
	OLS	OLS	OLS	OLS	2SLS	2SLS	OLS	OLS
*Panel A*	*(1)*	*(2)*	*(3)*	*(4)*	*(5)*	*(6)*	*(7)*	*(8)*
**Twin birth**	.668^***^	.671^***^	.035^***^	.021^***^				
**(IW)**	(.009)	(.009)	(.006)	(.005)				
IW > 2 children					.052^***^	.031^***^	.022^***^	.020^***^
at t + 6					(.009)	(.008)	(.002)	(.001)
Constant	.375^***^	.383^***^	1.427^***^	1.303^***^	1.408^***^	1.291^***^	1.419^***^	1.296^***^
	(.005)	(.009)	(.003)	(.006)	(.005)	(.006)	(.003)	(.006)
Adjusted R^2^	.054	.088	.020	.156	.019	.157	.021	.157
N	166,666	165,805	166,666	165,805	166,666	165,805	166,666	165,805
*Panel B*^a^	*(1)*	*(2)*	*(3)*	*(4)*	*(5)*	*(6)*	*(7)*	*(8)*
**Same Sex**	.043^***^	.043^***^	− .001	− .001				
**(IW)**	(.002)	(.002)	(.001)	(.001)				
IW > 2 children					− .026	− .026	.021^***^	.019^***^
at t + 6					(.035)	(.032)	(.002)	(.002)
Constant	.355^***^	.362^***^	1.428^***^	1.303^***^	1.437^***^	1.313^***^	1.420^***^	1.295^***^
	(.005)	(.009)	(.003)	(.006)	(.013)	(.013)	(.003)	(.006)
Adjusted R^2^	.027	.063	.020	.156	.016	.152	.021	.157
N	163,898	163,053	163,898	163,053	163,898	163,053	163,898	163,053
Other covariates^b^	No	Yes	No	Yes	No	Yes	No	Yes

Standard errors in parentheses. All specifications include dummies for mother’s age and calendar year of second birth

^a^ Women with twin births excluded in panel B

^b^ Years since 1st birth, Norwegian born, time since last move, employment, income, education, country region and centrality

^†^*p* < .1; ^*^p < .05; ^**^p < .01; ^***^p < .001

#### Main Results

The IV estimates for the family-friendliness of a mother’s final neighborhood show similar patterns as have been seen for the propensity to move: The sex-composition instrument gives a non-significant negative IV estimate (−0.03), while the twin IV estimate is positive but small (0.03 *p* < 0.01). In line with previous research, our OLS estimates (columns 7 and 8) show that index women’s high fertility is correlated with high historical fertility in their final neighborhood—and the estimate lies very close to that for the twin IV.

Parity specific results reveal that both the OLS and the twin IV estimates are largely driven by mothers of three children being more likely to live in neighborhoods with a large proportion of mothers with larger families (see Appendix Table A4). Hence, even though our previous analyses showed that increases in family size associated with twin births encouraged particularly high propensities to undertake short-distance consumption-related residential relocations, these same families also appear to end up in relatively family-oriented, high-fertility neighborhoods.

## Linking the Results

Fertility behavior is known to be correlated within neighborhoods, yet the relative importance of the mechanisms driving this correlation remains unclear. We used random variation in having a third child to test the explanatory power of two different mechanisms, namely selective moving behavior and social interaction effects among neighbors. To handle self-selection and confounding factors, we used the sex composition of the two eldest children and having twins at the second birth as instrumental variables (IVs) for family size increases.

When it comes to decisions regarding relocation, the OLS estimates show that third births are positively correlated with a family’s propensity to move. This is in line with previous studies that have consistently shown that births and residential relocations are closely related life course transitions (Ermisch & Steele, 2016; Feijten & Mulder, 2002; Kulu & Steele, 2013; Mulder, 2013; Öst, 2012).

Having twins at second birth raises the probability of a mother relocating, especially within short distances (less than three kilometers). The effect is present both in the short run (two years after the twin birth) and long run (after six years, the final year of observation). The immediate effects are concentrated among mothers who live in apartments and relatively smaller dwellings, suggesting adjustment moves due to increases in housing consumption. However, six years after their family increase these families also tend to live in neighborhoods with many children, and with particularly many other larger families. This is solid evidence that selective moves contribute to the residential clustering of fertility.

When the sex-composition IV was used, no main effects were identified in terms of propensity to move. We suggest two explanations for the diverging effects of the two instruments. First, the sex-composition IV captures the effects of third births due to a preference for having at least one child of each sex. Parents may, at some level, know that they are open to having a large family. This could lead them to locate to a spacious dwelling from the outset, so that they do not need to relocate when a third child is born. Second, the effects of the third child might be canceled out by the direct effects of the sex composition, for example in the form of more room-sharing among siblings of the same sex, as discussed in Sect. 3. However, also twins might share a room longer, and yet the estimated effects here are positive.

At the same time, the lack of similarity between the IV estimates indicates that at least one of the instruments provides limited information about the average third child in this setting. As regards a twin birth, we suspect that zero spacing between the second and third child means that the nature of the causal effect may be quite different from the average causal effect of a third child in the population. Next, it is well known that twin births are only conditionally random, but in this application, our instrument fares somewhat worse on tests for conditional randomness than what is ideal, potentially raising questions about the general validity of the instrument.

Turning to social interaction effects among neighbors, none of the instruments did show significant effects. More specifically, an index woman’s fertility increase did not influence the number of children of her original neighbors six years later. We note that the correlation between the index woman having a third child and her neighbors’ future number of children, as estimated by OLS, was also weak. Many women move after having children, and as neighbor relations thrive on proximity and everyday encounters, it is not clear whether initial neighbors keep contact after a move, and the extent of such contact. Hence, as long as families relocate, finding a study design that both excludes self-selection to neighbor networks and ensures the networks’ relevance seems especially difficult. As pointed out in previous sections, estimation is complicated by the fact that neighborhoods are not fixed entities, and fertility among neighbors may be correlated due to selective co-location and common environmental factors. Many studies on interaction effects among neighbors do not fully address selective moving behavior, at least not that of the other neighbors, which may lead to biased measures (Hedman, 2011; Hedman & van Ham, 2012). To avoid such bias, we have locked women to their neighborhoods before the year of the second birth (see Fig. 1 and Sect. 4.2), eliminating the possibility of correlated effects or self-selection. This restriction means that if women who have a third child move and then influence the fertility of their new neighbors, this will not be captured by our estimates. The need to lock neighbor networks to ensure ‘network exogeneity’ also means that we cannot exploit the full flexibility of time-varying and individual-centered neighborhoods that our data allow. As with any measurement error, failure to appropriately measure networks will bias estimates of network effects toward null.^11^

## Concluding Discussion

Taken together, our study demonstrates evidence that selective moves are an important driver of the residential clustering of large families. Such moves can follow an unanticipated fertility shock, such as having twins, but family size preferences can also influence housing choices before children are born. To the best of our knowledge, this has not been demonstrated previously with a design that handles selection bias as convincingly as we do here. We find little evidence of social interaction effects between neighbors, but we note that the measures we take to ensure causal identification potentially bias our measure of the effect toward null. Because of the difficulty of measuring social interaction effects among neighbors, we are reluctant to say that they do not exist, even though we did not identify them with our preferred design.

The focus of this study was on family size and third births, an important margin in the Norwegian context. We believe the results from using the twin IV might be transferable to other higher-order births in families who already have housing and some experience with kids. On the other hand, we do not believe the results to be equally applicable to the *transition* to parenthood. With its relatively high fertility and mobility, the Norwegian population served as case for our study. Due to Norway’s advantaged economic position and generous universalistic welfare state, individual families might be able to realize both their fertility and mobility desires to a larger degree than families in other European countries. At the same time, housing is an important budget post also for Norwegian families, and increasingly so. Hence, the economic constraints that families face when it comes to housing might be comparable across countries. Such economic constraints can influence decisions both about the number of children and where to live.

To conclude, the results presented in this paper identified selective moves as one plausible causal driver of residential clustering of large families. The effects we identify are relatively small, though statistically significant. This suggests that residential clustering of large families is also driven by factors that we effectively control for in our design—most importantly self-selection based on family-size preferences and a family-oriented lifestyle. Individuals with large families adjust their housing accordingly. Suitable housing stock for large families is not available everywhere, however, and not for every budget.^12^ As such, the need for larger housing contributes to the link between income and fertility. Providing suitable housing stock for larger families seems necessary for the creation of stable neighborhoods and communities for families and children (see also Mollborn et al., 2018; Wessel & Lunke, 2019). Where family-friendly housing is unavailable, expensive or in short supply, families might potentially change their fertility plans or experience a reduction in their quality of life if they have to choose between staying in inappropriate housing and moving out of their neighborhood.

Neighborhoods and residential adjustments are not typically in focus when discussing family policies (see, e.g., Bergsvik et al., 2021). In the light of puzzling fertility declines in the Nordic countries the last decade and a high interest in family-friendly policies in several countries, our study underscores the possibilities that lie in an examination of the influence of housing conditions and rising housing prices on childbearing (see also Sobotka et al., 2019). Some single studies have attempted to show how variations in rents and real estate market prices over time and between areas have affected fertility (e.g., Dettling & Kearney, 2014, Lovenheim & Mumford, 2013, Simon & Tamura, 2009). Yet, there is still room for more research identifying the causal linkages to family behavior. Housing has had little focus in family policies so far, while its relevance repeatedly is demonstrated in population research. There are several ways through which policies affect and regulate the real estate market and the building stock in central areas, potentially some that in future research could be used for effect evaluation. Families self-select into local areas and neighborhoods, and this needs to be accounted for in policy making and planning, for instance, in terms of housing policies and/or planning related to childcare and schooling. This study has contributed to an understanding of this interrelationship of fertility and relocation, but also to the literature on social interaction effects related to fertility by testing the relevance of yet another network, namely that of neighbors.

### Supplementary Information

Below is the link to the electronic supplementary material.

Supplementary file (DOCX 127 kb)

## Appendix

See Tables

**Table A1:** Descriptive statistics for subsamples of neighbors (women aged 20–36 at start)

	All	Childless	One child	Two child
Average number of neighbors	29.4	14.5	5.9	6.4
Mean average distance (meters)	399	399	398	407
Median distance (meters)	136	136	133	138
N (index women)	54,787	54,755	54,475	53,461

A1,

**Table A2:** Effects of the index woman having a third child on initial young female neighbors’ average number of children in t + 6, by neighbor subgroups (twin IV, sex mix IV and OLS estimates)

	**Twin IV**		**Sex mix IV**^a^		**OLS estimate**	
	2SLS	2SLS	2SLS	2SLS	OLS	OLS
	(1)	(2)	(3)	(4)	(5)	(6)
**Childless female neighbors**						
IW > 2 children (t + 6)	- .005	- .009	.046	.062	.021***	.008**
	(.015)	(.014)	(.058)	(.055)	(.003)	(.003)
Constant	.824***	.780***	.804***	.754***	.814***	.774***
	(.008)	(.011)	(.022)	(.022)	(.006)	(.010)
Adjusted R^2^	.003	.041	.003	.035	.005	.041
N	54,755	54,485	53,813	53,550	54,755	54,485
**Female neighbors with 1 child**						
IW > 2 children (t + 6)	.019	.017	.000	.029	.033***	.011**
	(.019)	(.018)	(.074)	(.070)	(.004)	(.003)
Constant	1.811***	1.801***	1.819***	1.799***	1.805***	1.803***
	(.010)	(.014)	(.028)	(.028)	(.007)	(.013)
Adjusted R^2^	.003	.047	.001	.047	.003	.047
N	54,475	54,208	53,538	53,278	54,475	54,208
**Female neighbors with 2 children**						
IW > 2 children (t + 6)	- .011	- .013	− .004	.009	.029***	.013***
	(.015)	(.015)	(.061)	(.058)	(.003)	(.003)
Constant	2.348***	2.379***	2.345***	2.372***	2.333***	2.369***
	(.008)	(.012)	(.023)	(.023)	(.005)	(.010)
Adjusted R^2^	− .001	.038	.000	.040	.003	.040
N	53,461	53,192	52,533	52,271	53,461	53,192
Other covariates^b^	No	Yes	No	Yes	No	Yes

Standard errors in parentheses. All specifications include dummies for index woman’s age and calendar year of second birth

^a^Women with twin births excluded in columns 3 and 4

^b^Years since 1^st^ birth, Norwegian born, time since last move, employment, income, education, country region and centrality

^†^*p* < .1; ^*^*p* < .05; ^**^*p* < .01; ^***^*p* < .001

A2,

**Table A3:** Effects of a having a third child on propensity to relocate with a distance of at least 3 km (twin IV and OLS estimates)

	**Twin IV**		**OLS estimate**	
	2SLS	2SLS	OLS	OLS
**Move > 3 km**	(1)	(2)	(3)	(4)
> 2 children (t + 6)	−0.010	0.009	0.078***	0.065***
	(0.014)	(0.014)	(0.003)	(0.003)
Constant	0.407***	0.451***	0.373***	0.429***
	(0.008)	(0.011)	(0.005)	(0.009)
Adjusted R^2^	0.008	0.072	0.015	0.075
N	166,927	166,063	166,927	166,063
Other covariates^a^	No	Yes	No	Yes

Standard errors in parentheses. All specifications include dummies for mother’s age and calendar year at second birth

^a^Years since 1st birth, Norwegian born, time since last move, employment, income, education, country region, and centrality

^***^*p* < .001

A3 and

**Table A4:** Effects of having a third child on family sizes in the final neighborhood, percentage of neighbors with at least one, two and three children (twin IV and OLS estimates)

	**Twin IV**		**OLS estimate**	
	2SLS	2SLS	OLS	OLS
	(1)	(2)	(3)	(4)
**Neighbors with ≥ 1 child**				
IW > 2 children (t + 6)	1.295***	.817***	-.385***	-.067
	(.316)	(.305)	(.057)	(.056)
Constant	65.772***	61.672***	66.420***	62.015***
	(.169)	(.236)	(.116)	(.212)
Adjusted R^2^	0.004	0.075	0.009	0.076
**Neighbors with ≥ 2 children**				
IW > 2 children (t + 6)	1.704***	1.009***	.464***	.491***
	(.338)	(.319)	(.061)	(.059)
Constant	48.557***	43.591***	49.035***	43.792***
	(.181)	(.248)	(.124)	(.221)
Adjusted R^2^	0.009	0.110	0.011	0.111
**Neighbors with ≥ 3 children**				
IW > 2 children (t + 6)	1.517***	.864***	1.432***	.969***
	(.238)	(.210)	(.044)	(.040)
Constant	18.176***	15.467***	18.208***	15.426***
	(.127)	(.163)	(.091)	(.145)
Adjusted R^2^	0.030	0.237	0.030	0.237
N	166,657	165,796	166,657	165,796
Other covariates^a^	No	Yes	No	Yes

Standard errors in parentheses. All specifications include dummies for mother’s age and calendar year of second birth

^a^Years since 1st birth, Norwegian born, time since last move, employment, income, education, country region, and centrality

^†^*p* < .1; ^*^*p* < .05; ^**^*p* < .01; ^***^*p* < .001

A4, Figs. A1 and A2.

## Footnotes

1 However, the use of various fertility treatments, such as IVF, has given rise to a correlation between twin births and features of mothers other than age alone. We return to this discussion in relation to the balancing tests in Sect. 4.5.

2 In the analyses, we only employ data on women, although partner characteristics, partnership status and potential changes in such statuses are also important. As there is sparse evidence to the effect that partner characteristics or the tendency of partnerships to dissolve differs between couples who have twins or children of the same sex as opposed to other couples with young children in the household (Jena et al., 2011), this is unlikely to influence our results substantially.

3 Cumulative results for each of the years in between t + 1 to t + 6 for the first two outcomes can be found in Appendix Figure A1 and Appendix Figure A2.

4 In our sample, the median distance moved is 3.8 km and declines with the age of the second child. This fits well with findings from other contexts, i.e., the study of Ermisch and Steele (2016), where the median distance moved was three kilometers.

5 The neighborhood is defined using basic statistical units, which on average include 131 women.

6 Unfortunately, the housing data have about 20 percent missing values, where in particular dwellings that have not recently been sold on the housing market presumably lack detailed information.

7 Significance levels .1, .05 and .01 are still common in the IV literature. By the same token, low r2 values are a common problem in 2SLS estimation, reflecting the fact that the instrumental variable uses only a small part of the variation to obtain unconfounded estimates.

8 The estimates differ marginally between Panel A and B because mothers of twins (N = 2,771) are excluded from the sample of Panel B.

9 Estimates are also close to zero for moves of at least five and ten kilometers. The larger the distance moved, the more similar the moving behavior of mothers who recently had a second child—with or without a fertility shock (results upon request).

10 Note that among those in the smallest dwellings the effects of a third birth could be less pronounced if the dwelling is already not suitable for the second child, also for those in the comparison group.

11 It might seem intuitively more reasonable to focus on how one woman’s fertility decision is influenced by those around her, and not on how she influences the fertility of her neighbors. For the sake of comparison, we estimated our main model on a different sample, where we measure an index woman’s probability of having another child depending on her neighbors’ fertility. In such a setup, we did not find any significant social interaction effects either, and there is still the possibility that some of the neighbors have made a selective (non)move because of their (intention to have a) third child, compromising the exogeneity of neighborhood family size composition. We note that the inability to distinguish between selective moves and social interaction effects in the new neighborhood applies to several applications of the aggregated instrument (Maurin and Moschion 2009).

12 It is, however, not unlikely that a phenomenon such as the spatial clustering of large families entails both family-friendly areas that are most accessible for economically privileged families as well as areas where spacious housing is accessible for large families with fewer economic resources.
