# Supplementary material for: Explaining Residential Clustering of Large Families
Source: Eur J Popul. 2023 Apr 19;39(1):13. doi: 10.1007/s10680-023-09655-6 (PMC10115922; doi:10.1007/s10680-023-09655-6)
Supplement: Supplementary file 1 — Supplementary file (DOCX 127 kb) [file 10680_2023_9655_MOESM1_ESM.docx]

**Appendices**

| **Appendix Table A1** Descriptive statistics for subsamples of neighbors (women aged 20-36 at start) | | | | |
| --- | --- | --- | --- | --- |
|  | All | Childless | One child | Two child |
| Average number of neighbors | 29.4 | 14.5 | 5.9 | 6.4 |
| Mean average distance (meters) | 399 | 399 | 398 | 407 |
| Median distance (meters) | 136 | 136 | 133 | 138 |
| N (index women) | 54,787 | 54,755 | 54,475 | 53,461 |

| **Appendix Table A2** Effects of index woman having a third child on initial young female neighbors’ average number of children in t+6, by neighbor subgroups (Twin IV, sex mix IV and OLS estimates) | | | | | | |
| --- | --- | --- | --- | --- | --- | --- |
|  | **Twin IV** | | **Sex mix IV^a^** | | **OLS estimate** | |
|  | 2SLS | 2SLS | 2SLS | 2SLS | OLS | OLS |
| **Childless female** | (1) | (2) | (3) | (4) | (5) | (6) |
| **neighbors** |  |  |  |  |  |  |
| IW > 2 children (t+6) | -.005 | -.009 | .046 | .062 | .021*** | .008** |
|  | (.015) | (.014) | (.058) | (.055) | (.003) | (.003) |
| Constant | .824*** | .780*** | .804*** | .754*** | .814*** | .774*** |
|  | (.008) | (.011) | (.022) | (.022) | (.006) | (.010) |
| Adjusted R^2^ | .003 | .041 | .003 | .035 | .005 | .041 |
| N | 54,755 | 54,485 | 53,813 | 53,550 | 54,755 | 54,485 |
| **Female neighbors** | (1) | (2) | (3) | (4) | (5) | (6) |
| **with 1 child** |  |  |  |  |  |  |
| IW > 2 children (t+6) | .019 | .017 | .000 | .029 | .033*** | .011** |
|  | (.019) | (.018) | (.074) | (.070) | (.004) | (.003) |
| Constant | 1.811*** | 1.801*** | 1.819*** | 1.799*** | 1.805*** | 1.803*** |
|  | (.010) | (.014) | (.028) | (.028) | (.007) | (.013) |
| Adjusted R^2^ | .003 | .047 | .001 | .047 | .003 | .047 |
| N | 54,475 | 54,208 | 53,538 | 53,278 | 54,475 | 54,208 |
| **Female neighbors** | (1) | (2) | (3) | (4) | (5) | (6) |
| **with 2 children** |  |  |  |  |  |  |
| IW > 2 children (t+6) | -.011 | -.013 | -.004 | .009 | .029*** | .013*** |
|  | (.015) | (.015) | (.061) | (.058) | (.003) | (.003) |
| Constant | 2.348*** | 2.379*** | 2.345*** | 2.372*** | 2.333*** | 2.369*** |
|  | (.008) | (.012) | (.023) | (.023) | (.005) | (.010) |
| Adjusted R^2^ | -.001 | .038 | .000 | .040 | .003 | .040 |
| N | 53,461 | 53,192 | 52,533 | 52,271 | 53,461 | 53,192 |
| Other covariates^b^ | No | Yes | No | Yes | No | Yes |
| *Note*: Standard errors in parentheses. All specifications include dummies for index woman’s age and calendar year of second birth.  ^a^ Women with twin births excluded in columns 3 and 4.  ^b^ Years since 1^st^ birth, Norwegian born, time since last move, employment, income, education, country region, and centrality.  ^†^ p<.1; ^*^ p<.05; ^**^ p<.01 ^***^ p<.001 | | | | | | |

| **Appendix Table A3** Effects of a having a third child on propensity to relocate with a distance of at least 3 kilometers (Twin IV and OLS estimates) | | | | | |
| --- | --- | --- | --- | --- | --- |
|  | | **Twin IV** |  | **OLS estimate** |  |
|  | 2SLS |  | 2SLS | OLS | OLS |
| **Move > 3km** | (1) |  | (2) | (3) | (4) |
| > 2 children (t+6) | -0.010 | | 0.009 | 0.078*** | 0.065*** |
| Constant | (0.014)  0.407*** (0.008) | | (0.014)  0.451*** (0.011) | (0.003)  0.373*** (0.005) | (0.003)  0.429*** (0.009) |
| Adjusted R^2^ | 0.008 | | 0.072 | 0.015 | 0.075 |
| N | 166,927 | | 166,063 | 166,927 | 166,063 |
| Other covariates^a^ | No | | Yes | No | Yes |
| *Note*: Standard errors in parentheses. All specifications include dummies for mother’s age and calendar year at second birth.  ^a^ Years since 1^st^ birth, Norwegian born, time since last move, employment, income, education, country region, and centrality.  ^†^ p<.1; ^*^ p<.05; ^**^ p<.01; ^***^p<.001 | | | | | |

| **Appendix Table A4** Effects of having a third child on family sizes in the final neighborhood, percentage of neighbors with at least one, two and three children (Twin IV and OLS estimates) | | | | |
| --- | --- | --- | --- | --- |
|  | **Twin IV** | | **OLS estimate** | |
|  | 2SLS | 2SLS | OLS | OLS |
| **Neighbors with ≥ 1 child** | (1) | (2) | (3) | (4) |
| IW > 2 children (t+6) | 1.295*** | .817*** | -.385*** | -.067 |
|  | (.316) | (.305) | (.057) | (.056) |
| Constant | 65.772*** | 61.672*** | 66.420*** | 62.015*** |
|  | (.169) | (.236) | (.116) | (.212) |
| Adjusted R^2^ | 0.004 | 0.075 | 0.009 | 0.076 |
| **Neighbors with ≥ 2 children** | (1) | (2) | (3) | (4) |
| IW > 2 children (t+6) | 1.704*** | 1.009*** | .464*** | .491*** |
|  | (.338) | (.319) | (.061) | (.059) |
| Constant | 48.557*** | 43.591*** | 49.035*** | 43.792*** |
|  | (.181) | (.248) | (.124) | (.221) |
| Adjusted R^2^ | 0.009 | 0.110 | 0.011 | 0.111 |
| **Neighbors with ≥ 3 children** | (1) | (2) | (3) | (4) |
| IW > 2 children (t+6) | 1.517*** | .864*** | 1.432*** | .969*** |
|  | (.238) | (.210) | (.044) | (.040) |
| Constant | 18.176*** | 15.467*** | 18.208*** | 15.426*** |
|  | (.127) | (.163) | (.091) | (.145) |
| Adjusted R^2^ | 0.030 | 0.237 | 0.030 | 0.237 |
| N | 166,657 | 165,796 | 166,657 | 165,796 |
| Other covariates^a^ | No | Yes | No | Yes |
| *Note*: Standard errors in parentheses. All specifications include dummies for mother’s age and calendar year of second birth.  ^a^ Years since 1^st^ birth, Norwegian born, time since last move, employment, income, education, country region, and centrality.  ^†^ p<.1; ^*^ p<.05; ^**^ p<.01; ^***^ p<.001 | | | | |


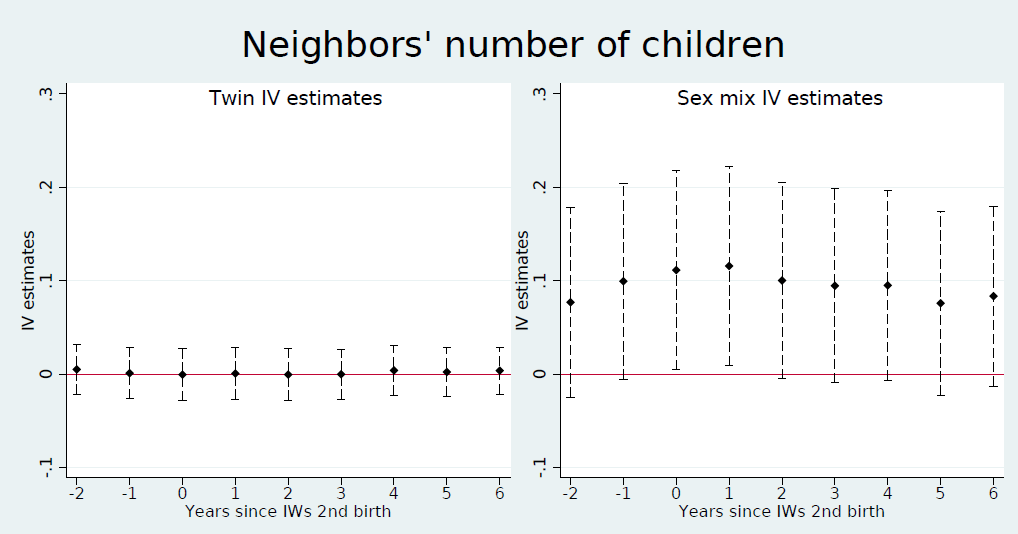


**Appendix Figure A1** Effects of index woman’s third child on initial young female neighbors’ average number of children (IV estimates with 90% CIs).


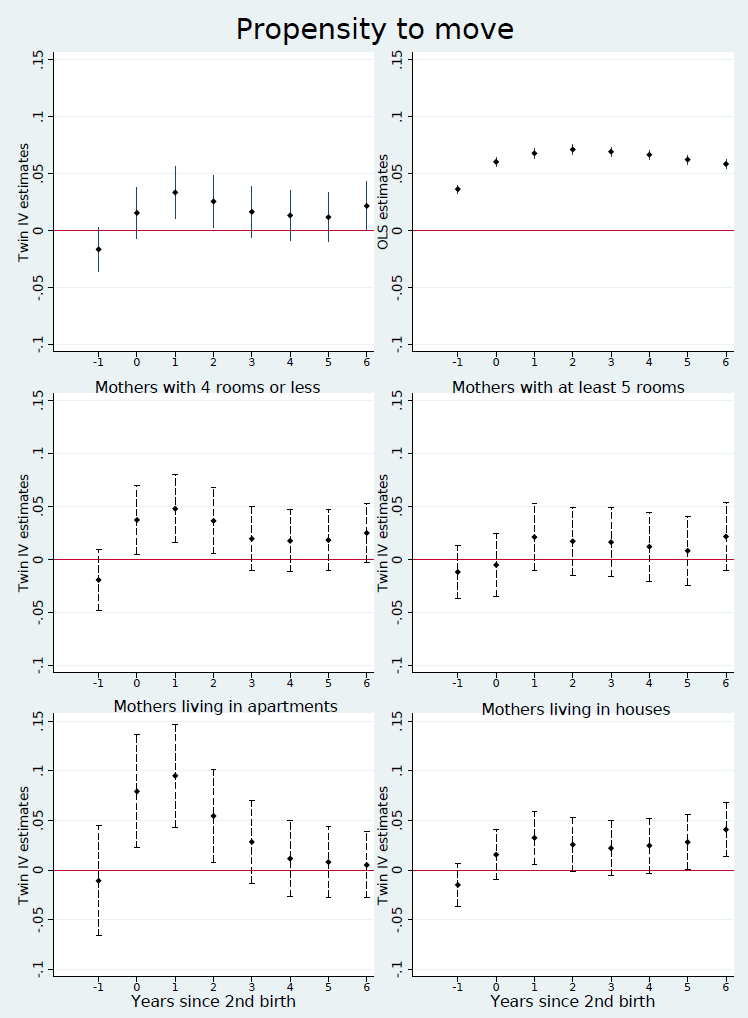


**Appendix Figure A2** Probability of having moved at least once before specified points in time, by size and type of dwelling (Twin IV estimates with 90% CIs).
